# The Psychology Analysis for Post-production of College Students’ Short Video Communication Education Based on Virtual Image and Internet of Things

**DOI:** 10.3389/fpsyg.2022.781802

**Published:** 2022-03-25

**Authors:** Wufeng Tang

**Affiliations:** ^1^Zhejiang University of Finance & Economics Dongfang College, Haining, China; ^2^Faculty Communication and Media Studies, Universiti Teknologi MARA (UiTM), Shah Alam, Malaysia

**Keywords:** virtual image, the Internet of Things, film and television education, college students, collaborative filtering

## Abstract

To improve the understanding of film and television postproduction for college students in the era of intelligent media, a study is conducted on college students’ short video communication education and audience psychology based on the rapid development of virtual image and the Internet of Things (IoT). Primarily, the collaborative filtering algorithm (CFA) is optimized and combined with the principle of Spark and Hadoop platforms as well as the IoT and virtual image technologies. Then, a hybrid computing model is proposed, and the two algorithms are improved and combined, with 90,000 network video records as data samples. Finally, the push accuracy of the hybrid algorithm and the traditional algorithm is calculated and compared, and based on this, a questionnaire survey on the audience psychology of short video production is carried out for college students. The results show that the time user of the combined algorithm is always at least 0.4 s faster than that of a single algorithm and the running speed of the algorithm with five nodes is nearly 80% higher than the algorithm with a single node. The Spark algorithm with multinode has good versatility in image recording and processing of large groups of college students. When processing more than 100,000 image records, the deviation values of Spark and Hadoop with a single node exceeded 1.1, but the deviation value of the hybrid algorithm was still lower than 1.1. With the increase of data volume, the deviation values of the three algorithms are increasing. Compared with the traditional CFA algorithm, the optimized algorithm has a higher speed in processing data and is more accurate in content pushing. From the questionnaire survey of college students, it is found that contemporary college students are not active in learning knowledge of virtual images. Hence, it is concluded that colleges must carry out relevant courses based on short video communication education and strengthen the short video communication education of college students. A reference is provided for the development of college students’ short video communication education in the digital age.

## Introduction

By improving the level of postproduction of film and television education for college students in the era of intelligent media and exploring the audience psychology of college students in the new era, this study introduces virtual image technology to study the state of college students in the field of film and television production. Digital development has not been realized in the postproduction of film and television education for college students at present. As new technologies, virtual image technology and the Internet of Things (IoT) technology are characterized by authenticity and coherence, which are very suitable for studying educational problems. Hence, a study is conducted on the postproduction of college students’ education and the audience psychology by virtual image and the IoT technology. With the rapid development of science and technology, people have changed from passively accepting information to actively seeking information, indicating that they are entering “the era of knowledge media” (Xu et al., 2019). A virtual image provides people with a variety of excellent quality in visual, auditory, technology, and artistic sense from time or space, so that virtual image is also known as the “the most potential art in the 21st century.” Virtual image education is an important part of art education in the new century. On the premise of improving the teaching environment, it can be accepted by most students, with timeliness and convenience (McReynolds et al., 2020). As the main carrier for college students to convey and receive information through virtual images, multimedia such as network movies, micro video, music, and multimedia news will produce a lot of data. Therefore, it has been a hot topic of the studies on how to combine virtual image technology with IoT technology to filter information and push them to college students (Su et al., 2020).

Virtual image is one of the main ways of information dissemination today. Various information is transmitted on the Internet to college students for their watching and listening. Zhang et al. (2020) used gray correlation and gray clustering algorithms combined with similar image vocabulary to determine the design board of gold jewelry, in which they understood the design image preferences of each group. Xue et al. (2021) used the prescribed selection method in the street design of the virtual environment for these streets to study public preference. He et al. (2021) expanded political education into a form of thought, proposed feasible strategies, and provided a reference for instructors and frontline workers in film and television education. Xie (2020) believed that virtual image was an artistic accomplishment and the preference of audience for film and television art laid a theoretical foundation for the quality and dissemination of film and television art. Wu et al. (2020) claimed that nurturing was usually in the early stages of development and that if someone wanted to develop the film and television industry, he must start with education and learning.

At present, the studies on the postproduction of film and television education for college students mainly focus on the theories of film and television education form, and there is a shortage of practical studies based on new technologies. There are also few studies on the combination of virtual images and IoT technology. The short video communication education production based on virtual image mainly chooses the virtual image pushing algorithm to conduct a study, but this algorithm is not intelligent enough and a single algorithm has certain limitations in dealing with problems. The studies of college students’ audience psychology under the background of new technology mainly focus on the acceptance of new technology. Therefore, the present work uses a hybrid algorithm to study the post-production and audience psychology of college students’ short video communication education to provide a reference for the development of college students’ film and television education in the new era. The content of the present work positively impacts college students in the era of intelligent media. A data pushing method is realized based on virtual images through the Hadoop and Spark collaborative filtering algorithms (CFAs) of the IoT. Besides, a comparison is made on the accuracy of the two methods of virtual image technology pushing and single pushing when college students make pictures through micro-video production or image processing tools on the daily network. Through the questionnaire survey, an analysis is conducted on the psychological attitude of the audience of film and television education to the virtual image of college students. Simultaneously, another analysis is made on the importance of film and television education for college students. The innovation is to analyze the accuracy of virtual image data pushing and the demand of college students for short video communication education. The present work has positive significance for the improvement of college students’ acceptance of short video communication education. It has an impact on the development of virtual image technology and IoT technology in the field of short video communication education for college students.

## Related Studies

Virtual image belongs to new media technology. New media art derives from the conceptual art of the 1960s, as well as early futuristic declarations, expressive acts, and the 1970s performing arts (Zhang, 2021). The German Media Art Center is the earliest and largest science and technology media art center in the world. Its goal establishment is to promote the development of media art and explore the impact of new technology on contemporary art. Other influential new media arts institutes include the Massachusetts Institute of Technology in the United States, the Tokyo Media Arts Center in Japan, and the Linz Electronic Arts Festival in Austria (Chen et al., 2021). The Dutch Media Art Center, which is located in Rotterdam, was founded in 1981. It is the earliest pioneer of contemporary media art, which concludes only a small group of young artists. Today, it has developed into an internationally renowned scientific and technological media art institution.

The history of the research and development of new media art in China is a process from imitation to creation. The initial imitation of new media art includes two levels, which are the imitation of traditional art and the imitation of foreign excellent new media artworks, respectively (Zhou et al., 2021). The development of new media art in China began in the late 1980s. It is the epoch-making of video works by Zhang Peili at the China Modern Art Exhibition in 1989, which is the first new media practice in the history of China’s new media art (Yu et al., 2021). Since then, artists’ attempts at new media have never stopped. The artistic activities of new media such as the Shanghai Biennale in China, the International New Media Art Exhibition and Forum, the Asia-Pacific Multimedia Art Festival, the China Independent Image Festival, and the Shanghai Electronic Art Festival have brought about many outstanding achievements in the artistic creation of new media, which reflects the active attempts of contemporary artists in the field of new media (Alhayani and Llhan, 2021). Although from the digital media technology, the current development in China is not lagging behind compared with that of foreign countries. However, in the expression of artistic language, there is still a big gap between the artistic works (Ma C. M. et al., 2021). The current new media art research in China mainly focuses on the field of art theory. There is a lack of research on digital virtual images and other new media technology application practices and services for commercial purposes case analysis and collation.

The Internet of Things technology emerged relatively late in China. Besides, there is not enough curriculum related to film and television education for college students and there is a lack of research literature on short video communication education. The number of publications has increased from 1 to 145 on short video communication education in China in the past 30 years, which shows that the current number is not considerable (Dong, 2021). Research on a contents-pushing algorithm by virtual image shows that the algorithm is not intelligent enough. The number of college students is numerous and their personal preferences are not the same. Faced with massive virtual videos, they waste a lot of useful resources. Hence, a single algorithm cannot meet the demand and a mixed contents-pushing algorithm is a feasible solution to this problem.

Yang (2019) commented that under the influence of micro video, film, and television education of China failed to achieve its teaching purpose. However, Western education has a long history and the education system is relatively complete (Yang, 2019). Tong et al. (2021) reckoned that socializing through virtual movies could also help companies gain recognition and trust from their partners. Yuan and Wu (2020) pointed out that the ability of learners to collaborate and coordinate in a team is becoming increasingly important for the success of any work and the advancement of knowledge. Hence, short video communication education is also very important for the cooperative inquiry ability of college students.

## Materials and Methods

### Virtual Image on the Networks in the Era of Intelligent Media

No longer limited to professionals, people with smart devices can be both the creators and publishers of virtual images or videos. From 2-h movies to tens of seconds of micro videos, the works based on virtual image technology appear with a spurt of development. Because the IoT is real-time, shared, and diverse, college students have become the main force of virtual image dissemination and creation. They can choose or produce virtual film and television works anytime and anywhere on the Internet through various channels. The increase of video publishers on the network is directly related to the increase in college students’ video consumption.

**FIGURE 1:**
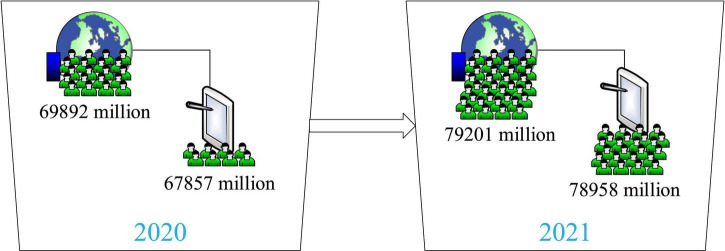
Network video user scale.

According to the statistical report on network development released by the Information Center in 2021, by 2020, the number of network users in China has reached nearly 790 million (Wahab et al., 2020), as Figure 1. Compared with 2019, it increased by nearly 100 million, among which mobile phone users accounted for 98%. The report pointed out that compared with other apps, online video apps used 12.8% longer (Ma G. et al., 2021). Therefore, online video based on virtual images has become an important part of the lives of people.

The virtual image is based on digital technology, combined with visual and auditory communication technologies, which include many kinds of technologies, such as photography, projection, multimedia display, sensory transmission, limb movement capture, and imitation (Bing and Qian, 2021). Virtual images are provided for users through devices to help people understand abstract concepts. It concludes with interactive images and scenes composed of words, images, videos, and other information. Its main features are interactivity, virtualization, and immersive experience (Hudson et al., 2019).

**FIGURE 2:**
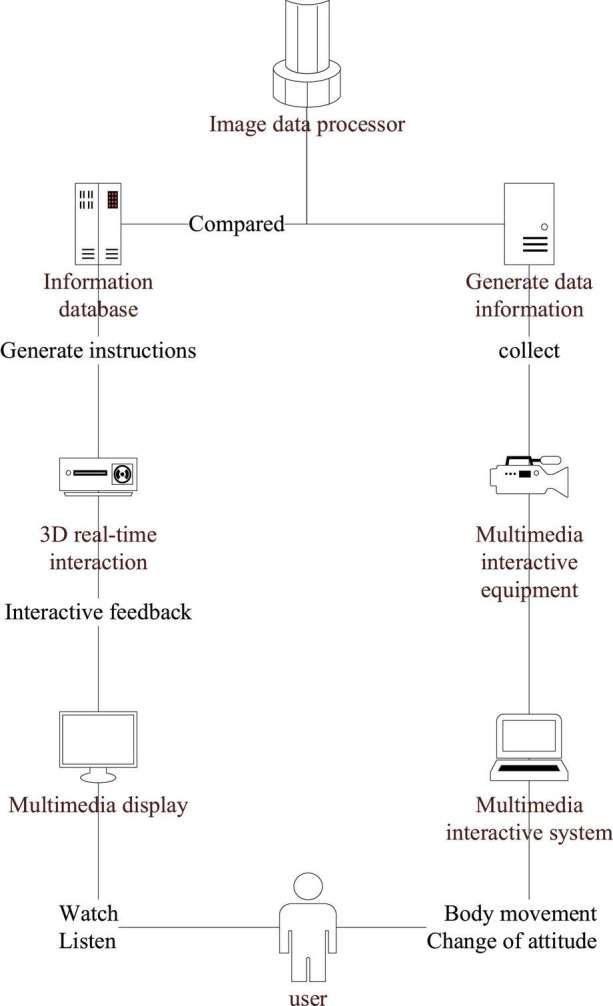
Virtual technology [virtual reality (VR)] helmet principle.

The most common virtual image technology is virtual reality (VR) technology (Wohlgenannt et al., 2020). VR is a technology made by modeling software (3DMAX, Unity3D), which can provide users with an omnidirectional observation environment (Hsiao, 2021). At present, VR is used in immersive teaching, games, simulation, and other industries (Stojšić et al., 2019). However, VR equipment is mainly in the form of a pair of glasses or a helmet and its working principle is shown in Figure 2 (Hoppe et al., 2021). When the equipment is being used, the visual field of users will be closed, so it has certain limitations.

**FIGURE 3:**
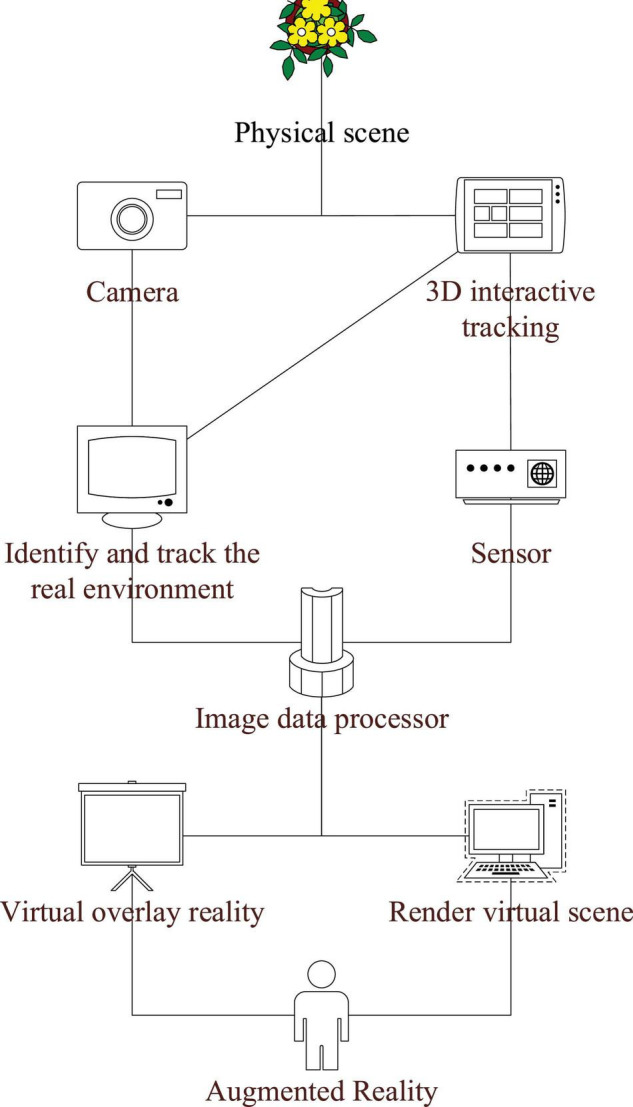
Augmented reality (AR) principle of reality enhancement technology.

Reality enhancement technology augmented reality (AR), that is, all kinds of information in life (Radosław and Katja, 2021). AR technology generally uses cameras to identify and analyze observed things and matches them with images and three-dimensional (3D) models in the database of the IoT. For sound information, it displays the screen output through digital information and uses sensing technology and 3D tracking as the principle, as shown in Figure 3. The set gestures are used to control the device and interact with the virtual environment (Chen et al., 2019). AR technology usually includes such technologies as data identification, environment display, image fusion, sensor blending, real-time tracking, etc. It has strong interactivity and has a good development prospect in the fields of teaching, product design, art design, museum display, tourism promotion, etc (Sarigoz, 2019).

**FIGURE 4:**
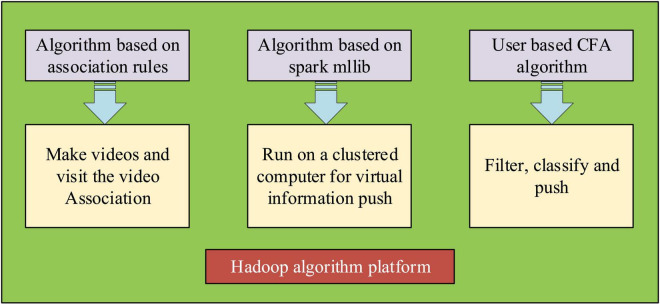
The overall layout of the algorithm.

The present work uses the algorithms based on association rules, Spark MLlib, the user-based CFA, and Hadoop, as shown in Figure 4. The content of specific algorithms is given in the following sections.

### Association Rules and Spark MLlib Algorithm

(1) Algorithm based on association rules (Francisco and Jon, 2021). In the operation, the video or image information visited by the user is analyzed as the main body. When the user makes the video or image in the later stage, the virtual algorithm is used to push the relevant video to the device. The purpose of the rule is to make the video and visit the video association. At present, the most typical example is the hobby recommendation of various video websites. By analyzing the access history or the access frequency influenced by a certain video, another media file with high similarity is pushed to the equipment of the users.
(2) Algorithm based on Spark MLlib. Under the background of the continuous development of Spark technology, the IoT algorithm technology called Spark MLlib is widely used in machine learning algorithms (Hung et al., 2019) and the algorithm is usually run in cluster computers.

**FIGURE 5:**
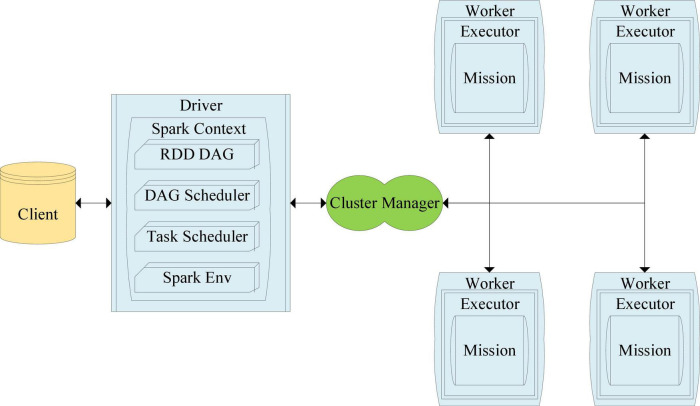
Spark algorithm flow.

Among them, the MLlib algorithm by Spark, which can be used as an alternating least squares algorithm (Xu and Li, 2019), has been widely used in the virtual information push system. The alternating least square (ALS) algorithm models the establishment of the score evaluation matrix as the multiplication of low-order user U and virtual information item V factor and then simplifies and minimizes the reconstruction error of the observed score. After analyzing these factors, images or graphics can be made or recommended according to the predicted scores. Figure 5 illustrates the execution flow of the Spark algorithm.

**FIGURE 6:**
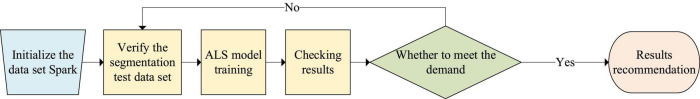
The alternating least square (ALS) algorithm flowchart.

The main idea of the ALS algorithm is not to predict by analyzing similarity with common user’s favorite article recommendation rules, but to calculate by decomposing matrix. Figure 6 displays the structural flow of the ALS algorithm.

### User-Based Collaborative Filtering Algorithm

Due to the ceaseless development of the commerce industry in the electronic field, the variety and quantity of commodities in commerce are increasing and very complicated data are produced in the process of circulation. Therefore, an algorithm for screening, classifying, and then pushing data is urgently needed.

The CFA (Fan et al., 2019) is the most popular algorithm in various systems at present. On the basis of the near technical scheme, the distance between users is analyzed, and the access or evaluation of a certain thing is obtained by the neighbor user with the smallest distance between users to judge the degree of preference of a certain user for a certain item. Thus, more related data can be put to this user in the form of virtual images. The CFA also has some flaws. Some popular videos or images on the Internet cannot be pushed accurately, while college students recommend these videos or images to each other. Videos with more visits will be pushed to user devices after algorithm statistics, which lead to such recommendations having no practical significance.

**FIGURE 7:**
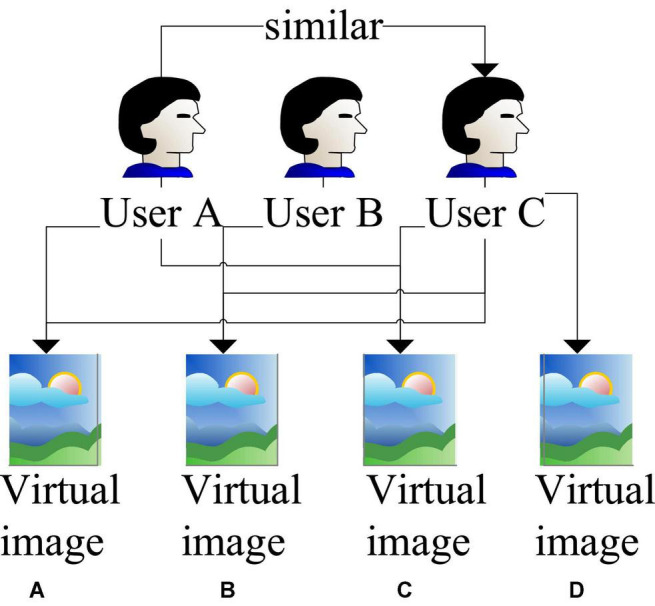
Algorithm principle based on user collaboration. **(A)** Group A video materials. **(B)** Group B video materials. **(C)** Group C video materials. **(D)** Group D video materials.

The main research purpose of this algorithm is to analyze the degree of students’ interest in accessing virtual images in the daily network. For example, when a certain group of students with the same or similar interests visit online videos and images or make referencing materials in the postproduction, there is a certain degree of similarity in the items used. The tool A used by user A will also be the tool frequently used by user B. The material A frequently referenced by user A will also be the material frequently used by user D. Figure 7 shows the basic principle of this algorithm.

In the current research, there are two rules for calculating user access similarity: one is the cosine similarity and the other is the modified cosine similarity.

The first is cosine similarity (Dominik et al., 2021): when studying the reference production degree of a certain virtual image by college students, analysis is generally made by this score evaluation matrix, and the vectors in the n-dimensional space are set as the score evaluation matrix. Under the default condition, “0” indicates the image that has not been evaluated by scores, and the similarity degree is analyzed by the cosine angle between the vectors. The results can be similar. Assuming that vector i and vector j, respectively, represent the scores of user i and user j in n-dimensional space, the studied university student user i and university student user j are obtained and the similarity calculation can be made between them as eq. (1).


(1) $$\cos\left(i,j\right)=\frac{i\cdot j}{||i||\cdot ||j||}$$

(2) M=UTV*

In eq. (1), u represents the matrix of order m. Parameter t refers to m × n order matrix. v stands for the n-order matrix. The regularization matrix decomposition equation is:


(3) $$m\,i\,n_{U,V}\,\sum_{\left(u,i\right)\in K}{\left(R_{u,i}-U_{u}^{T}\,V_{i}\right)}^{2}+\lambda \,\left({||U_{u}||}^{2}+{||V_{i}||}^{2}\right)$$

K is the set of fractions (u and i), and *R*_*u,i*_ refers to the true evaluation score of user u for image i. λ represents the regularization coefficient and ||*U*_*u*_||^2^ + ||*V*_*i*_||^2^ stands for a regularization project. Set the score evaluation matrix r to be m × n matrix, with U and V, respectively, and the score can be represented by U and V as eq. (4):


(4) $$R_{u,i}^{\wedge }=U_{u}^{T}\,V_{i}$$

The second is the modified cosine similarity (Bakri and Hashim, 2018): there are some drawbacks in cosine similarity calculation, and there is no detailed analysis of the corresponding relationship between the size of the score evaluation scale and the degree of interest of college students in images. Assuming that the evaluation is scored from 1 to 5 points, for user A, the score of 4 points may represent a high degree of interest, but for user B, 3 points may also mean that he has a high degree of interest in a certain image. The essence of this problem lies in the different evaluation standards among individual users. However, because this standard exists at all times, in order to remove the influence of this standard on the evaluation results and improve its drawbacks, two sets of vectors are set. $R_{i}^{-}$ and $R_{j}^{-}$, respectively, represent the average scores of users i and j in the N-dimensional space. Therefore, eq. (2) is the similarity calculation equation between users i and j.


(5) $$\cos\left(\text{i}-R_{i}^{-},\text{j}-R_{j}^{-}\right)=\frac{\left(i-R_{i}^{-}\right)\cdot \left(j-R_{j}^{-}\right)}{||i-R_{i}^{-}||\cdot ||j-R_{j}^{-}||}$$

Based on the above two groups of equations, a similar production reference of the virtual images of users can be obtained. This method can analyze the results of daily visits to images by target university students. It is assumed that N*^k^*_*u*_ represents the combined group of K neighbors of user U and user U evaluates the score of image J. *R*_*u,i*_, calculated as (6).


(6) $$R_{u,i}=R_{i}^{-}+\frac{\Sigma_{v\in N_{u}^{k}}\,\sin\left(u,v\right)\cdot \left(R_{v,i}-R_{v}^{-}\right)}{\Sigma_{v\in N_{u}^{k}}\,\sin\left(u,v\right)}$$

### Hadoop Algorithm

**FIGURE 8:**
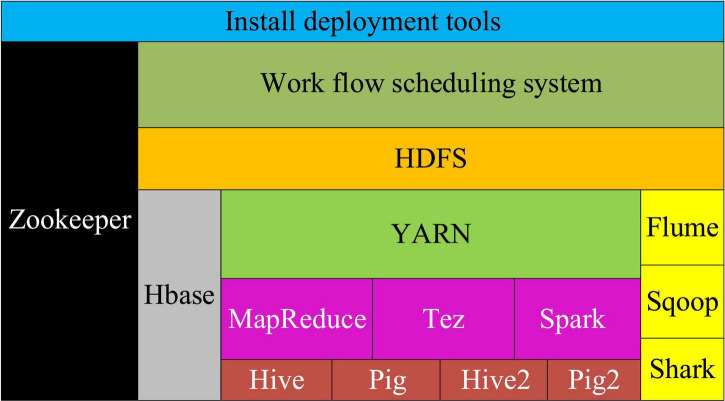
Hadoop structure.

Hadoop algorithm (Zeebaree et al., 2020) is an algorithm based on distributed computing with ease of use, which can run on the Hadoop platform and process a larger amount of data. Among the currently widely used data analysis algorithms, Figure 8 signifies the specific structural system.

Hadoop algorithm has the following advantages:

High availability: Using bit-by-bit storage not only ensures the overall system processing efficiency, but also the ability to process massive data is equally efficient.

Efficient: Dynamic data can be moved between nodes, which ensures that the computing nodes are in an efficient running state and also ensures the dynamic balance of the overall load.

High scalability: With the Hadoop algorithm, data distribution is performed in available computing clusters, which is convenient to extend to many nodes.

High fault tolerance: With the Hadoop algorithm, the data copy is automatically saved, and the failed tasks are redistributed to ensure the normal operation of the system, without affecting the overall task processing due to the downtime of a computer.

Low cost: Hadoop algorithm is an open-source and efficient processing technology and compared with data processing technology, it does not need a lot of money.

**FIGURE 9:**
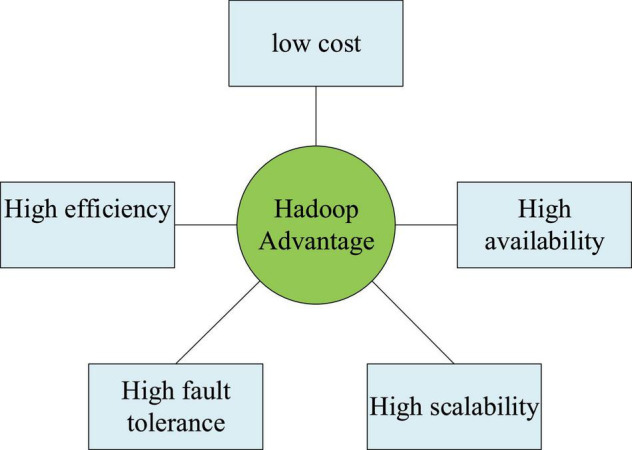
Advantages of the Hadoop algorithm.

Figure 9 shows the summarized advantages.

### College Students’ Audience Psychology of Short Video Communication Education

**FIGURE 10:**
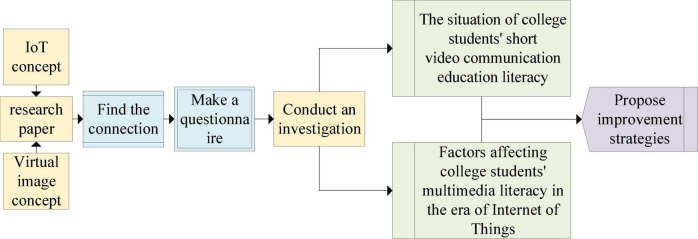
Investigation process.

According to the development of virtual images in the era of intellectual media, a questionnaire is designed for the audience psychology of short video communication education for college students. After relevant literature is studied, Figure 10 is used to explicate the flow of the survey.

The questionnaire is developed to investigate the problems from three aspects, namely, (1) personal information of students, (2) literacy of short video for college students, and (3) school examination-oriented education. Table 1 shows the specific information of the questionnaire.

**TABLE 1:** Questionnaire of film and television education for college students.

Your gender: A. Male, B. Female
Your grade: A. freshman, B. sophomore, C. junior, D. senior
Your major: A. Literature, B. Economics, C. Science, D. Medicine E. Law, f. Art, g. Agriculture, H. Philosophy I. Management, J. Education, K. History, L. Others
What do you think of the role of film and television works? A. broaden your horizons, B. relax and entertain, C. kill time, D. course needs
You know something about virtual images: A: Not at all, B. Not at all, C. General, D. A little understanding, E. Very understanding
Will you evaluate after watching the film and television works? A. Never, B. Rarely, C. Generally, D. Occasionally, E. Always
Channels for you to obtain information about film and television: A. Books, B. Network, C. Recommended by others
What are your concerns about film and television works? A. actor, B. plot, C. special effects, D. director, e. editing
Do you think college students need short video communication education? A. Not at all, B. Not much need, C. General, D. More need, E. More need
Is there a short video communication education course in your school? A. Yes, B. No
If yes, what do you think of the course: A course is rich in connotation and of great significance, B. Talking is better than nothing, but it is optional, C. It is boring and completely useless
Other suggestions for film and television education:

Since the research contents are mainly based on virtual images and the IoT, college students are taken as a whole to analyze the influence on the postproduction of college students’ short video communication education. Therefore, there are few studies on the influence of descriptive factors and the survey of the present work only focuses on the gender of college students.

The research object is college students from a university. A total of 300 questionnaires are distributed and 294 questionnaires are recovered. Among them, eight questionnaires are invalid (damaged and the survey questions are not answered), 286 questionnaires are counted, and the effective rate of the questionnaire is 95.3%. There are 129 males and 157 females and the proportion of males and females is relatively balanced. The SPSS version 26.0 software (SPSS Incorporation, Armonk, NY, United States) is used for the analysis of the simple correlation of the data in the recovered questionnaires.

The reliability test table of the questionnaire is determined by Cronbach’s α. If the reliability of the sample data is greater than 0.7, it means that the items in the scale are consistent and the test index is reasonable. The validity is tested by the Kaiser–Meyer–Olkin (KMO) value. If the KMO value is greater than 0.7, the validity is good, which proves that the questionnaire design is reasonable and effective.

## Results

### Experimental Environment

All the experimental settings are based on the experimental results and relevant rules. Table 2 lists the experimental environment.

**TABLE 2:** Experimental environment.

Items	Names	Specific contents
Hardware	Lipids	ROM 2G, Genuine Intel(R) CPU T1350 @1.86 GHz
Software	Linux	Ubuntu 12.04; Fedora 10
Development environment	Eclipse	

### Dataset Collection and Data Preprocessing

The dataset is from the Internet image records of the region where the research object is located, with a total of 100,000 image records. 100,000 image records are used in the combination of the Spark algorithm and Hadoop algorithm. In the accuracy analysis on contents-pushing, five groups of data are randomly selected, namely, 10,000, 30,000, 50,000, 70,000, and 90,000. The same five groups of data are used in the operating rate analysis of systems with multinode and multidata. The data are from 286 questionnaires and they are analyzed by the SPSS version 26.0 software (SPSS Incorporation, Armonk, NY, United States).

The algorithm analysis uses Microsoft Excel for preliminary screening of data, so there is no data preprocessing. The questionnaire is directly analyzed by the SPSS version 26.0 software (SPSS Incorporation, Armonk, NY, United States), so there is no need for data preprocessing.

### Parameters Setting

The important parameter settings of the Hadoop platform are introduced: file:/// is set as the default value of fs.defaultFS, the parameters are interpreted as system host and port, 4096 is set as the default value of io.file.buffer.size, the parameter is interpreted as the buffer size of the stream file, /tmp/hadoop-${user.name} is set as the default value of hadoop.tmp.dir, and the parameters are interpreted as temporary folders.

### Performance Metrics

The performance index in the system rate analysis of Spark algorithm combined with Hadoop algorithm in the present work is the node running speed; the performance index of push accuracy analysis based on virtual image records is the deviation between the actual value and the predicted value; the performance index in the system operation rate analysis of multinode and multidata is the image data processing efficiency; the performance index of the questionnaire survey results of college students’ film and television education is the proportion of people.

### Performance Evaluation and Discussion

#### Spark Combined With Hadoop System Rate Analysis

**FIGURE 11:**
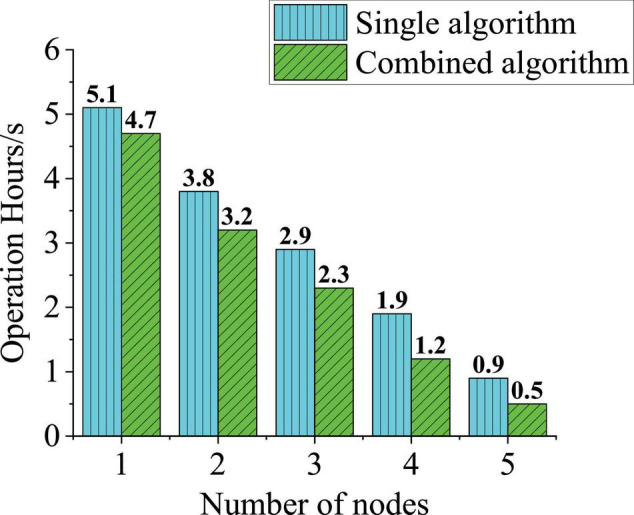
Image data analysis time.

A total of 100,000 image records are used to analyze the running efficiency of the system and a single algorithm and combined algorithm are used to count the running time of push virtual image data and the running speed of the system with 1–5 nodes is analyzed in total. Figure 11 shows the experimental results.

Through analyzing the data, Figure 11 signifies that with the increase of data nodes, the running time of the two algorithms is decreasing, but the time of combining algorithms is always at least 0.4 s faster than that of a single algorithm and the running speed of 5 nodes is nearly 80% higher than that of a single node. Because the multinode processing is compared with the single-node processing, in the Spark algorithm, the main node will manage and adjust other nodes. Multiple tasks are assigned to different nodes for processing, so that the processing speed of image data can be improved at the same time. Therefore, the multinode Spark algorithm has good universality in image recording processing for a large group of college students. The Spark algorithm has a great ability to process images. The research of Roghani et al. (2021) on the Spark algorithm showed that the image processing performance of the Spark algorithm was very excellent and the results of the present work were basically consistent with the latest research results.

#### Analysis of Push Accuracy Based on Virtual Image Records

**FIGURE 12:**
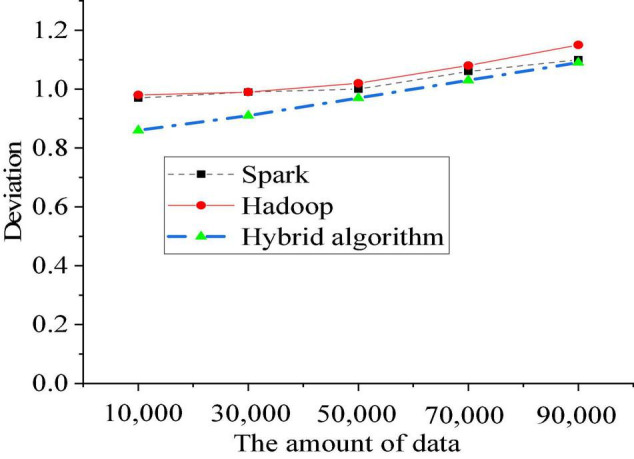
Deviations of different algorithms.

In this step, five nodes are used for analysis, and five groups of data are used for statistics, namely, 10,000, 30,000, 50,000, 70,000, and 90,000. The push accuracy is analyzed by combining the Spark, Hadoop, and double algorithms. The analysis results use the deviation value between the actual value and the predicted value to explain the accuracy. The average value is calculated as the result through the algorithm. Figure 12 shows the experimental results.

Figure 12 shows that among the three processing methods, the deviation value of the hybrid algorithm is the lowest. When processing more than 100,000 image records, the deviation value of Spark and Hadoop has exceeded 1.1. Among them, the deviation value of the Hadoop algorithm is the highest, reaching 1.2, but the deviation value of the hybrid algorithm is still lower than 1.1. As the number of data increases, the deviation values of the three algorithms increase. But, the deviation growth rate of hybrid algorithms is generally less than the other two single algorithms. Rezaeipanah et al. (2021) proved that the performance of the hybrid algorithms was often higher than that of single algorithms due to the shortcomings between algorithms, which was consistent with the conclusion of the present work. Therefore, the hybrid algorithm has high accuracy in pushing virtual image data according to the personal preferences of the user among large groups of college students. The algorithm can be used for video push in the later stage of college students’ short video communication education. Pushing accuracy is an important indicator of video push in the later stage of college students’ film and television education.

#### System Operation Rate of Multinode and Multidata

**FIGURE 13:**
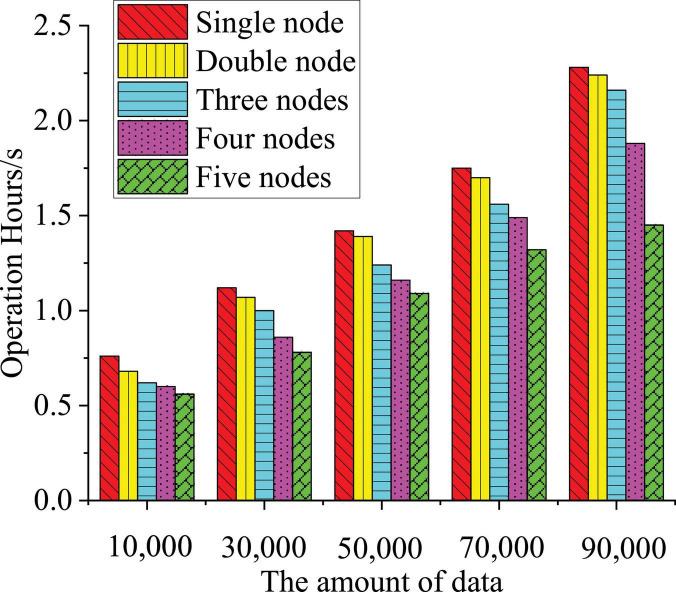
Data processing rates of different nodes.

In this section, five image data processing methods (from one node to five nodes) are used to analyze the processing rate of five groups of data (from 10,000 to 90,000), with the purpose of studying the image data processing efficiency of different nodes with increasing data volume. Figure 13 shows the results.

Through data comparison, it can be found that when dealing with less data, both the single node and multinode can efficiently deal with it, but with the increase of data volume, the processing rate of a single node gradually decreases. When the data volume is 100,000, the running time has exceeded 2.2 s, while the multinode remains below 1.5 s. In fact, the amount of college students’ image data far exceeds 100,000. Therefore, the speed of a single node is not enough to meet the requirements. Generally, the multinode algorithm is the best choice for massive image data. The latest research of Gilanifar and Parvania (2021) on multinode algorithm indicated that multinode algorithm was widely used in image processing research and helped to get good results. The present work also draws a conclusion that multinode algorithm has excellent application in image processing (Gilanifar and Parvania, 2021). The analysis shows that the multinode processing method is the most suitable image processing method for the massive postproduction images of college students’ short video communication education with multinodes.

#### College Students’ Film and Television Education Questionnaire Results

**FIGURE 14:**
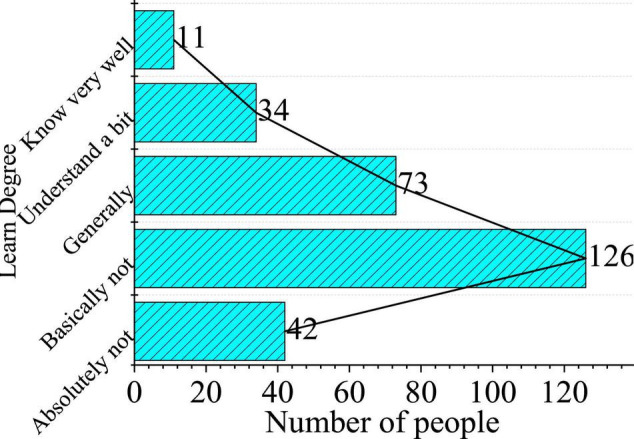
Knowledge level of a virtual image.

The reliability value of the present work is 0.83 and the validity value is 0.86, so the reliability and validity are good. The results of the questionnaire show that gender and image knowledge have no significant effect on the cognitive level of college students. Therefore, according to all the survey results, some representative questions are selected for statistical analysis. Figure 14 manifests the statistical analysis of students’ knowledge of virtual images.

The results show that 168 out of 286 people do not understand it, accounting for 58%, only a few people know it very well, and quite a few students do not understand it at all, which shows that college students learn less about the knowledge and concepts of virtual images. Therefore, it is necessary for colleges to offer short video communication education courses to relevant students. This provides data support for the research motivation. The latest research of Makransky and Petersen (2021) on the virtual image cognition of college students signified that the cognition of virtual images of college students was not deep enough (Makransky and Petersen, 2021). According to the research, the present work makes a quantitative expression of the results.

**FIGURE 15:**
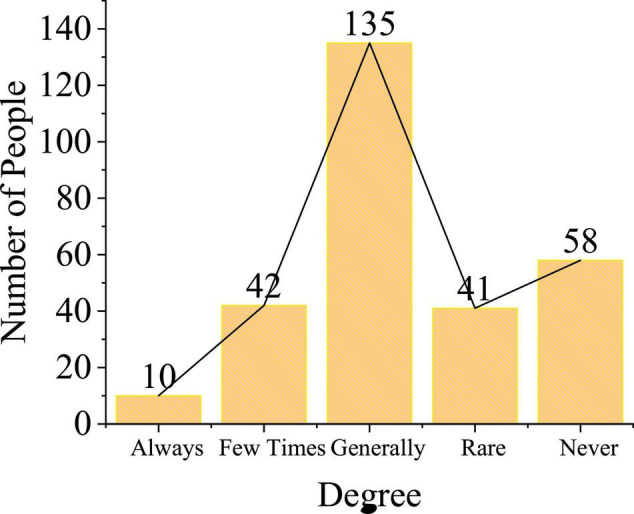
Release of video review.

An evaluation survey is published on video images. Whether the students will publish their evaluation after watching the video shows the attitude of the students toward short video and also reflects their own literacy of short video, as shown in Figure 15.

The initiative of students to publish the evaluation of videos reflects their own literacy of short video. Higher literacy of short video shows that the school attaches great importance to short video communication education. However, from the results, only 52 people who account for 18% will evaluate short video and the rest of the students rarely publish them, which indicates that the school does not attach great importance to film and television education for college students. The research of Xu (2021) on short video communication education for college students under the current network situation explored that Chinese college students paid less attention to film and television education (Xu, 2021). Although this conclusion is not directly drawn by the present work, the accuracy of this conclusion is confirmed. Therefore, it is necessary to carry out short video communication education courses in local colleges. Film and television education will provide strong guidance for the improvement of the postproduction ability of college students.

## Conclusion

In the era of informative media, college students should adopt more advanced technologies to master short video knowledge. Colleges should carry out short video communication education courses based on the IoT and virtual image. The IoT should push the virtual video to the target according to the daily video traffic to help students have rich material in the later stage of virtual video production. Based on the Spark and Hadoop algorithms, the present work proposes an algorithm to push related content according to virtual image records. Through experimental comparison, it is found that in the case of multiple nodes, the combined algorithm is superior to the single algorithm mode in data processing rate and video push accuracy. By using the questionnaire survey method, the present work investigates the current situation of short video communication education for students and finally summarizes the importance of contemporary short video communication education for college students. It is found that college students have little knowledge and concept of virtual images. Schools pay little attention to short video communication education for college students, so it is necessary to carry out short video communication education courses in local colleges and universities. The limitation is that the algorithm only focuses on the research of push accuracy and push speed and does not conduct in-depth research on the selection criteria. There is no more investigation on the personal information of investigators in the questionnaire setting including age, educational background, family background, etc. Therefore, it will be analyzed in the future. A reference is provided for the development of short video communication education for college students in the era of intelligent media. This research is expected to be applied in the short video communication education classroom for college students, to improve college students’ mastery of film and television knowledge under the new era and new technology, and promote the effective integration of virtual image technology and college students’ short video communication education.

## Data Availability Statement

The raw data supporting the conclusions of this article will be made available by the authors, without undue reservation.

## Ethics Statement

The studies involving human participants were reviewed and approved by the Zhejiang University of Finance and Economics Ethics Committees. The patients/participants provided their written informed consent to participate in this study. Written informed consent was obtained from the individual(s) for the publication of any potentially identifiable images or data included in this article.

## Author Contributions

The author confirms being the sole contributor of this work and has approved it for publication.

## Conflict of Interest

The author declares that the research was conducted in the absence of any commercial or financial relationships that could be construed as a potential conflict of interest.

## Publisher’s Note

All claims expressed in this article are solely those of the authors and do not necessarily represent those of their affiliated organizations, or those of the publisher, the editors and the reviewers. Any product that may be evaluated in this article, or claim that may be made by its manufacturer, is not guaranteed or endorsed by the publisher.
